# Full-length fruit transcriptomes of southern highbush (*Vaccinium sp.*) and rabbiteye (*V. virgatum* Ait.) blueberry

**DOI:** 10.1186/s12864-022-08935-5

**Published:** 2022-10-29

**Authors:** Yi-Wen Wang, Savithri U. Nambeesan

**Affiliations:** 1 Department of Horticulture, University of Georgia, 1111 Miller Plant Sciences Building, 120 Carlton Street, Athens, GA 30602 USA; 2 Center for Applied Genetic Technologies, University of Georgia, 111 Riverbend Road, Athens, GA 30602 USA

**Keywords:** Fruit quality, Fruit genomics, Postharvest, Ripening, Transcriptome

## Abstract

**Background:**

Blueberries (*Vaccinium sp.*) are native to North America and breeding efforts to improve blueberry fruit quality are focused on improving traits such as increased firmness, enhanced flavor and greater shelf-life. Such efforts require additional genomic resources, especially in southern highbush and rabbiteye blueberries.

**Results:**

We generated the first full-length fruit transcriptome for the southern highbush and rabbiteye blueberry using the cultivars, Suziblue and Powderblue, respectively. The transcriptome was generated using the Pacific Biosciences single-molecule long-read isoform sequencing platform with cDNA pooled from seven stages during fruit development and postharvest storage. Raw reads were processed through the Isoseq pipeline and full-length transcripts were mapped to the ‘Draper’ genome with unmapped reads collapsed using Cogent. Finally, we identified 16,299 and 15,882 non-redundant transcripts in ‘Suziblue’ and ‘Powderblue’ respectively by combining the reads mapped to Northern Highbush blueberry ‘Draper’ genome and Cogent analysis. In both cultivars, > 80% of sequences were longer than 1,000 nt, with the median transcript length around 1,700 nt. Functionally annotated transcripts using Blast2GO were > 92% in both ‘Suziblue’ and ‘Powderblue’ with overall equal distribution of gene ontology (GO) terms in the two cultivars. Analyses of alternative splicing events indicated that around 40% non-redundant sequences exhibited more than one isoform. Additionally, long non-coding RNAs were predicted to represent 5.6% and 7% of the transcriptomes in ‘Suziblue’ and ‘Powderblue’, respectively. Fruit ripening is regulated by several hormone-related genes and transcription factors. Among transcripts associated with phytohormone metabolism/signaling, the highest number of transcripts were related to abscisic acid (ABA) and auxin metabolism followed by those for brassinosteroid, jasmonic acid and ethylene metabolism. Among transcription factor-associated transcripts, those belonging to ripening-related APETALA2/ethylene-responsive element-binding factor (AP2/ERF), NAC (NAM, ATAF1/2 and CUC2), leucine zipper (HB-zip), basic helix-loop-helix (bHLH), MYB (v-MYB, discovered in avian myeloblastosis virus genome) and MADS-Box gene families, were abundant.

Further we measured three fruit ripening quality traits and indicators [ABA, and anthocyanin concentration, and texture] during fruit development and ripening. ABA concentration increased during the initial stages of fruit ripening and then declined at the Ripe stage, whereas anthocyanin content increased during the final stages of fruit ripening in both cultivars. Fruit firmness declined during ripening in ‘Powderblue’. Genes associated with the above parameters were identified using the full-length transcriptome. Transcript abundance patterns of these genes were consistent with changes in the fruit ripening and quality-related characteristics.

**Conclusions:**

A full-length, well-annotated fruit transcriptome was generated for two blueberry species commonly cultivated in the southeastern United States. The robustness of the transcriptome was verified by the identification and expression analyses of multiple fruit ripening and quality–regulating genes. The full-length transcriptome is a valuable addition to the blueberry genomic resources and will aid in further improving the annotation. It will also provide a useful resource for the investigation of molecular aspects of ripening and postharvest processes.

**Supplementary Information:**

The online version contains supplementary material available at 10.1186/s12864-022-08935-5.

## Background

Blueberry (*Vaccinium sp.*) is in the *Ericaceae* family and native to North America. Blueberries are gaining popularity due to increased awareness of their health benefits, such as lowering the risk of cardiovascular diseases and damage due to aging [1, 2]. In 2020, its total utilized production was 637 million pounds, which included fresh market fruit at 350 million pounds and fruit for processing at 288 million pounds [3]. In the United States, cultivated blueberries ranked fifth after grape, apple, strawberry, and sweet cherry in the value of utilized production in the non-citrus fruit and nuts category totaling 904 million dollars [3]. There are several important cultivated blueberry species in the United States. They vary in their cold hardiness and chill hour requirements for flowering. Lowbush (*Vaccinium angustifolium* Ait.) and northern highbush (*Vaccinium corymbosum* L.) are grown mainly in the northern US, while rabbiteye (*V. virgatum* Ait.) and southern highbush (hybrids of *V. corymbosum, V. virgatum, V. darrowii* Camp.) are predominantly cultivated in the southern US [4, 5].

To facilitate blueberry breeding, it is important to generate and enhance genetic and genomic resources. Originally a draft blueberry genomic sequence was generated using a diploid *V. corymbosum* accession W8520 [6]. Subsequently RNA-Seq reads from fruit developmental stages were assembled onto this draft blueberry genome, which predicted around 60,000 gene models. Of these, 58% were functionally annotated by homologous protein search and around 24% were assigned GO terms [7]. Subsequently an improved version of the haploid-phased northern highbush blueberry genome was generated from a tetraploid northern highbush cultivar ‘Draper’, which predicted 32,140 protein coding genes per haplotype. The GO annotation for this genome was around 57%, which was a significant improvement from the previous draft genome. More recently, a reference genome for *Vaccinium darrowii*, an evergreen wild blueberry has been generated [8, 9]. However, there is still no available genome for southern highbush or rabbiteye blueberries, those usually grown in the southeastern parts of the US.

Transcriptome, RNA expressed from the genome, is an invaluable resource for genome assembly and annotation [10], linking genes to their function [11], and is an alternative resource when the complete genome is not available [12]. Pacific Biosciences (PacBio) single-molecule long-read isoform sequencing (Iso-seq) is a method for full-length transcript sequencing without the need for further assembly [13]. This approach can provide accurate information of transcript structure and alternative splicing. In fruit crops, this sequencing technology has been used to identify potential genes related to disease resistance in apple [14], proanthocyanidin accumulation in persimmon [15], carotenoid biosynthesis in avocado [16], and sugar-metabolism in *Annona squamosa* [17]. In this study, we developed the first full-length fruit transcriptome for two blueberry cultivars, a tetraploid southern highbush ‘Suziblue’ and hexaploid cultivar ‘Powderblue’. In addition, to evaluate the robustness of the fruit transcriptome, we measured three fruit ripening and quality-related traits (ABA and anthocyanin concentrations, and texture) and the trait-related gene expression by qRT-PCR based on the transcriptome sequences. This work will provide additional valuable genomic resources that can be exploited by the blueberry community.

## Results

### Transcriptome sequencing and annotation

**Fig. 1:**
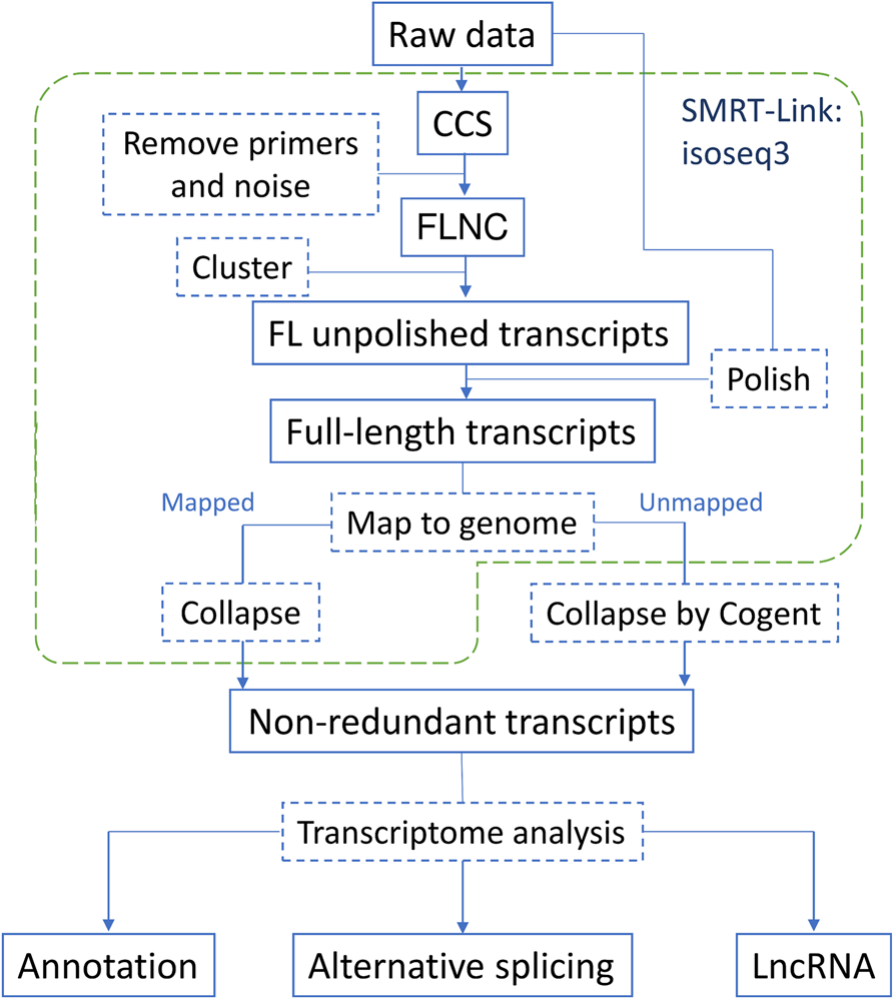
Flow chart for reconstruction of fruit specific and full-length transcriptomes in southern highbush blueberry ‘Suziblue’, and rabbiteye blueberry ‘Powderblue’ with Iso-Seq. CCS: circular consensus sequences, FLNC: full-length, non-concatemer reads, Cogent: Coding genome reconstruction tool, LncRNA: long non-coding RNA

**Fig. 2:**
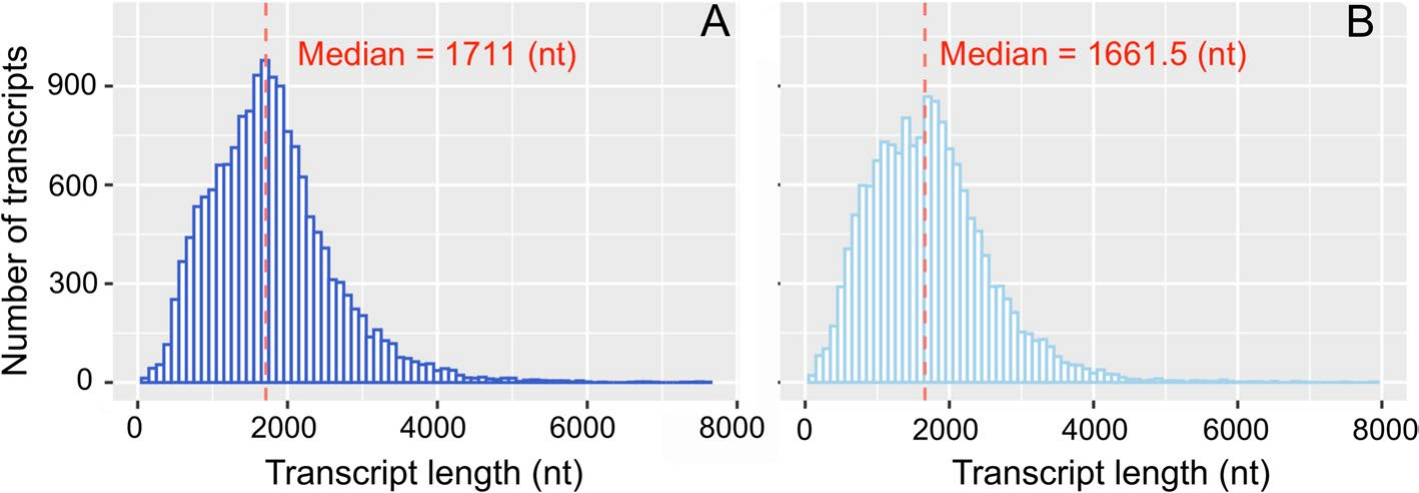
The distribution of non-redundant transcripts’ length in the fruit transcriptome in ‘Suziblue’ southern highbush (**A**) and ‘Powderblue’ rabbiteye blueberry (**B**)

Iso-Seq generated 29.48 Gb raw reads in ‘Suziblue’ and 25.82 Gb in ‘Powderblue’ (Table 1). Downstream analyses by the SMRT-Link Isoseq3 pipeline (Fig. 1), produced 541,220 circular consensus sequences (CCS) in ‘Suziblue’ and 482,718 CCS in ‘Powderblue’ (Table 1). This resulted in 31,846 high quality and 273 low quality full-length transcripts in ‘Suziblue’, and 31,091 high quality and 232 low quality full-length transcripts in ‘Powderblue’. Next, both high- and low-quality full-length transcripts were mapped to the ‘Draper’ genome to reduce redundancy of transcripts (Fig. 1). These analyses resulted in mapping of 89% of ‘Suziblue’ and 92% of ‘Powderblue’ full length transcripts to the ‘Draper’ genome. The remaining unmapped transcripts were collapsed by Cogent (Fig. 1). Non-redundant transcripts generated after mapping to the Draper genome and Cogent were combined (Table 1). Ultimately, 16,299 and 15,882 non-redundant transcripts were identified in ‘Suziblue’ and ‘Powderblue’ respectively (Table 1). The distribution of the lengths of non-redundant transcripts ranged from 75–7,650 nucleotides (nt) in ‘Suziblue’ and 60–7,945 nt in ‘Powderblue’ (Fig. 2). More than 80% of sequences have length greater than 1,000 nt, and the median length of the transcripts was around 1,700 nt in both cultivars (Fig. 2).

**Table 1:** Summary of raw reads, circular consensus sequences, and non-redundant transcripts of fruit transcriptomes in ‘Suziblue’ southern highbush and ‘Powderblue’ rabbiteye blueberry

Cultivar	Raw reads (Gb)	Circular consensus sequences (No.)	Non-redundant transcripts (No.)
Suziblue	29.48	541,220	16,299
Powderblue	25.82	482,718	15,882

**Fig. 3:**
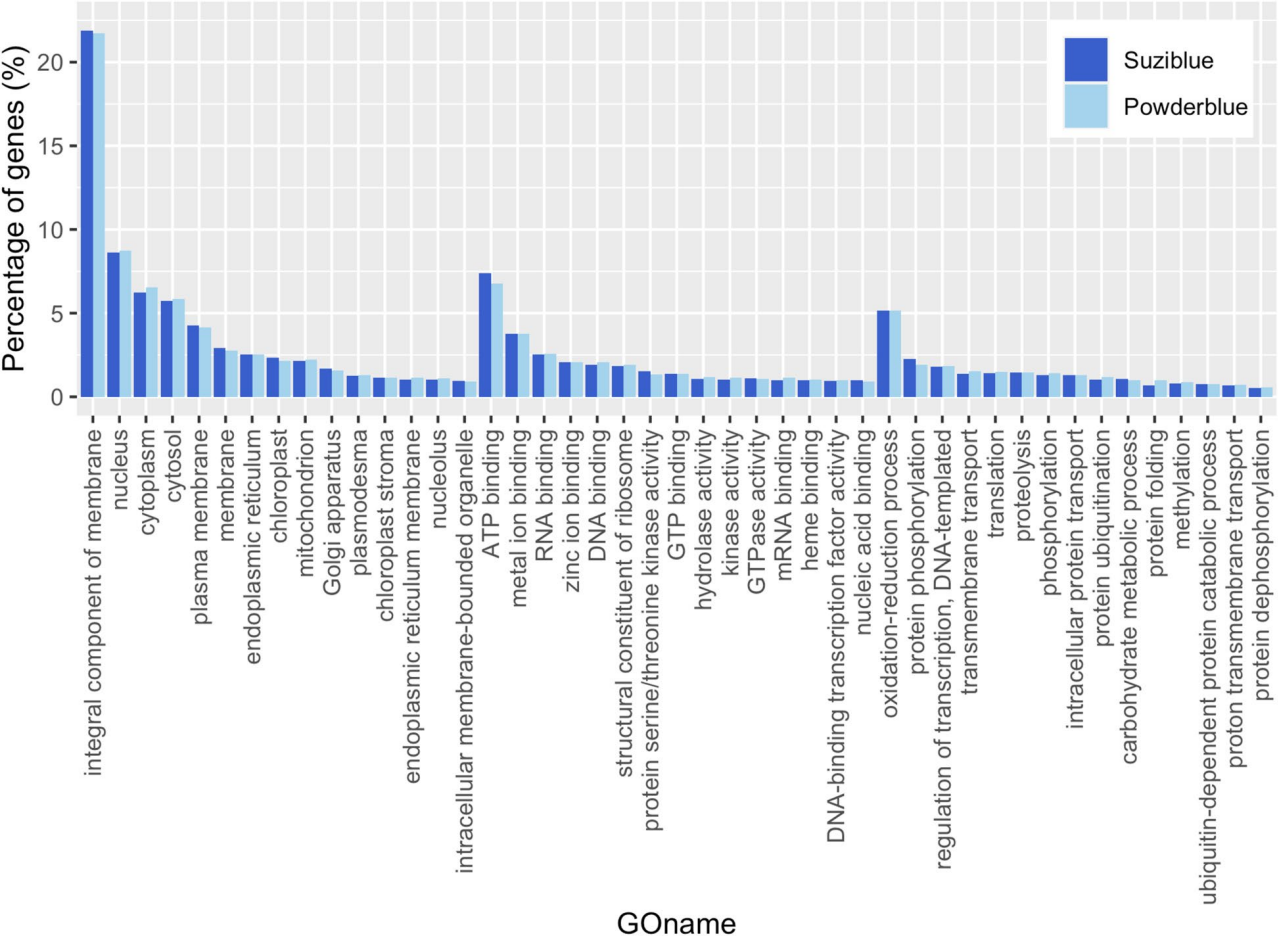
Gene ontology annotation of the fruit transcriptomes for ‘Suziblue’ southern highbush and ‘Powderblue’ rabbiteye blueberry. The annotation was conducted by Blast2GO with Blastx searching against NCBI non-redundant protein database with flowering plants as taxonomy filter

The non-redundant transcripts were subsequently annotated by Blast2GO. There were 15,172 and 12,734 transcripts that were functionally annotated by performing blastx against NCBI non-redundant protein database in ‘Suziblue’ and ‘Powderblue’ respectively (Table 2, Additional file 1: Table S1, S2). The functionally annotated transcripts were greater than 92% in both cultivars (Table 2). Additionally, 78% of these transcripts have at least one GO annotation, and more than 60% of transcripts have a GO term associated with cellular component, molecular function, and biological process (Table 2). Overall, the distribution of the GO terms in ‘Suziblue and ‘Powderblue’ are similar (Fig. 3). The top categories in cellular, molecular and biological processes were integral component of membrane, ATP binding, and oxidation reduction process respectively (Fig. 3).

**Table 2:** The number and the percentage of the functional annotated transcripts for the fruit transcriptomes by Blast2GO in ‘Suziblue’ southern highbush and ‘Powderblue’ rabbiteye blueberry

Annotation		Suziblue	Powderblue
Functional annotated		15,172 (93%)	12,734 (92%)
GO annotated	Cellular component	9,767 (60%)	9,533 (60%)
	Molecular function	10,388 (64%)	10,082 (63%)
	Biological process	9,905 (61%)	9,587 (60%)
	Total	12,734 (78%)	12,394 (78%)

### Alternative splicing analysis

**Fig. 4:**
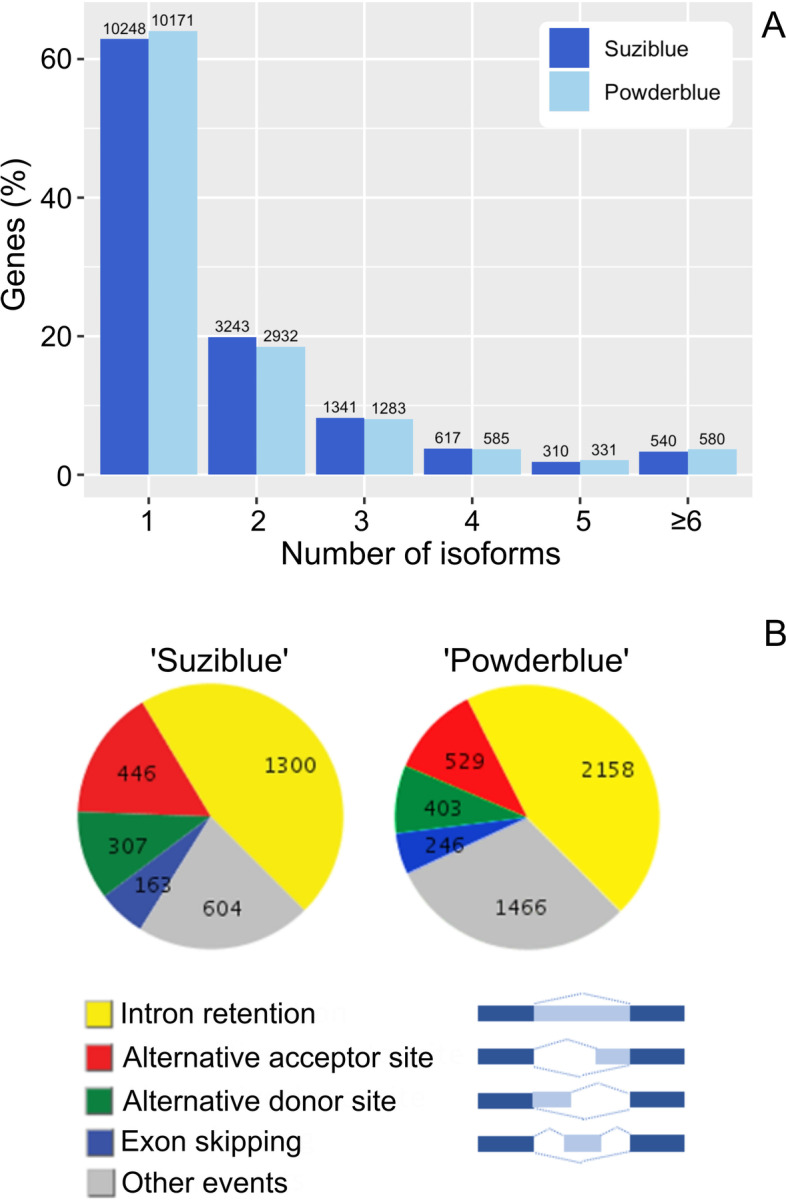
Alternative splicing analysis of ‘Suziblue’ southern highbush and ‘Powderblue’ rabbiteye blueberry with Iso-Seq fruit-specific full-length transcripts. **A** Distribution of the number of isoforms for each non-redundant transcript is presented. The number above each bar indicates the number of non-redundant transcripts. **B** Identification of alternative splicing events by AStalavista. Contribution of various pathways to generation of alternatively spliced transcripts s presented

Overall, the number of isoforms' distributions were similar in ‘Suziblue’ and ‘Powderblue’ (Fig. 4A). Approximately 60% of non-redundant sequences had no additional isoforms, while 40% had more than one isoform, suggesting alternative splicing (Fig. 4A). About 20% of the non-redundant sequences had 2 isoforms, and about 3.5% had more than 6 isoforms (Fig. 4A). Further, intron-retention was the most common alternative splicing event, which was 46% in ‘Suziblue’ and 45% in ‘Powderblue’ (Fig. 4B). The other alternative splicing events in the descending order were alternative acceptor site, alternative donor site, and exon skipping (Fig. 4B).

### Long non-coding RNA analysis

**Fig. 5:**
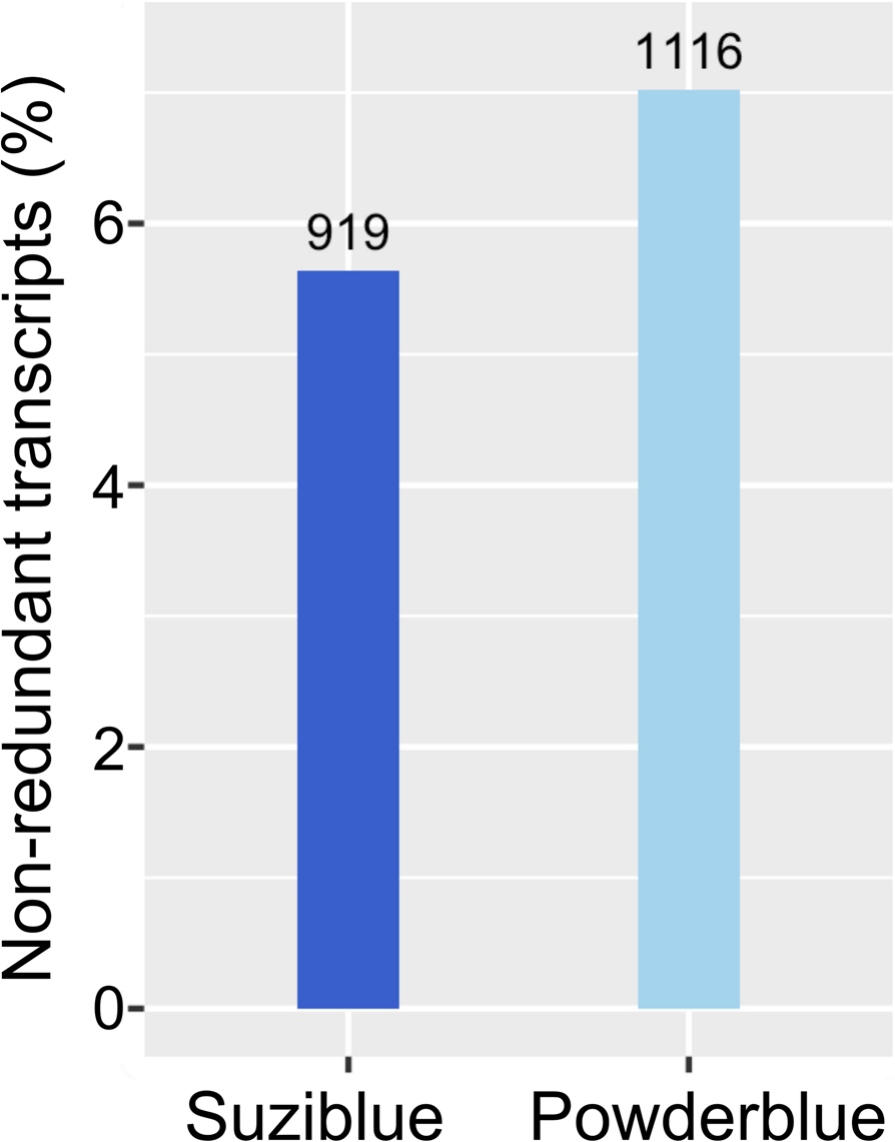
Long non-coding RNA in the fruit transcriptomes of ‘Suziblue’ southern highbush and ‘Powderblue’ rabbiteye blueberry. Analyses were performed using the Coding-Non-Coding Identifying tool. The proportion of long non-coding RNA within the non-redundant transcripts is presented

There were 919 and 1,116 transcripts predicted as long non-coding transcripts with a length longer than 200 nt in ‘Suziblue’ and ‘Powderblue’ (Fig. 5, Additional file 1: Table S3). These constituted 5.6% and 7% the transcripts respectively in the two genotypes (Fig. 5).

### Phytohormone-related and TF abundance in the transcriptomes

**Fig. 6:**
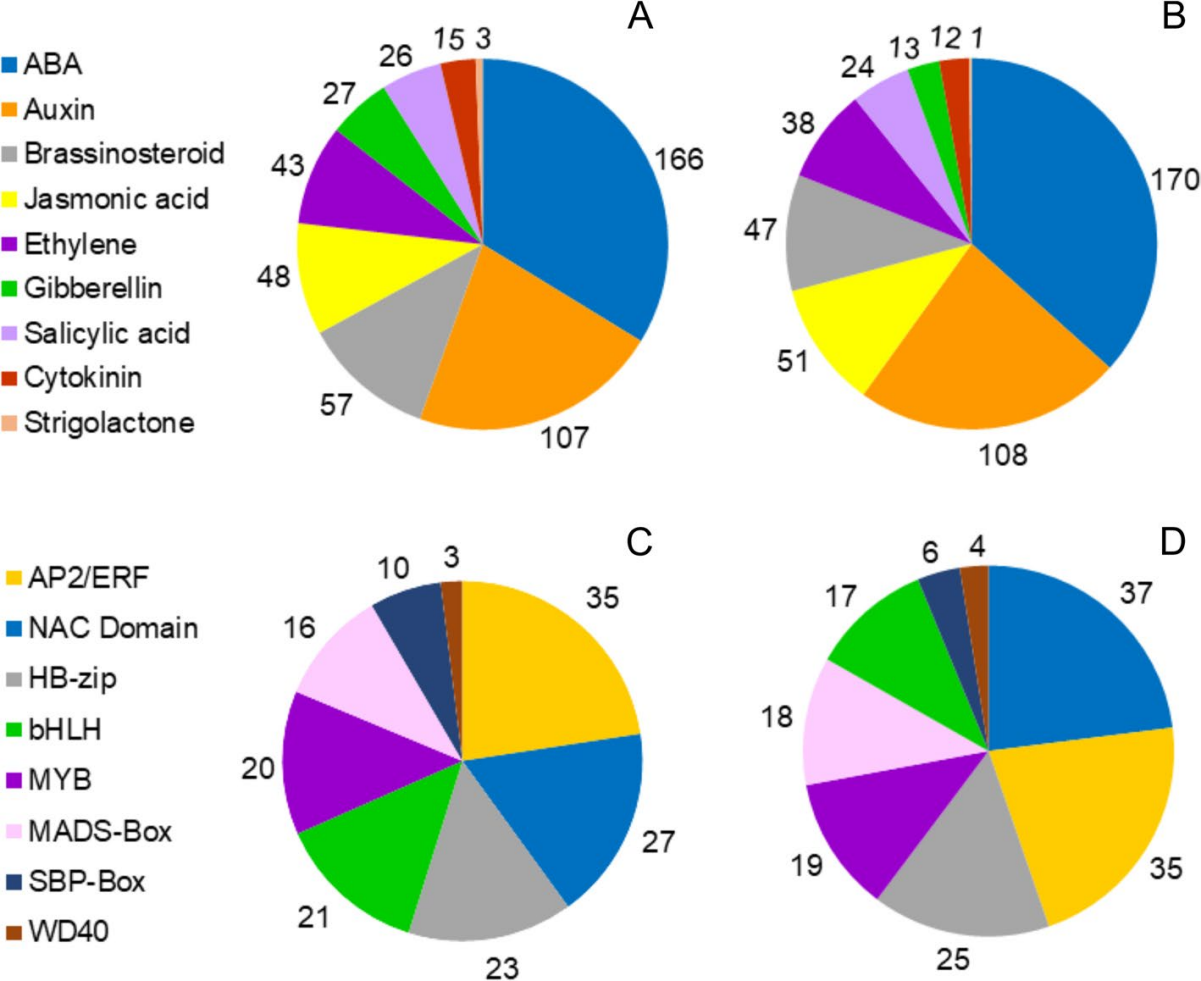
Summary of the number of transcripts belonging to hormone categories (**A, B**) and transcription factor families in (**C, D**) Suziblue (**A, C**) and Powderblue (**B, D**). For genes related to phytohormones, the GO category for biological processes was searched using hormone-related key words. For transcription factors, sequences from tomato and strawberry were retrieved from the Sol Genomics Network and NCBI, and their homologs in blueberry transcriptome were identified using BLAST analysis (tblastx function)

Overall the number and distribution among various phytohormone categories between the two cultivars were similar (Fig. 6A, B). The highest number of transcripts were related to ABA and auxin metabolism in both cultivars (Fig. 6A, B). The transcripts in these categories were assigned to response to hormones or hormone-activated/mediated signaling pathway (Additional file 1: Table S4, S5). These were followed by transcripts related to brassinosteroids and jasmonic acid metabolism and subsequently by transcripts associated with ethylene metabolism (Fig. 6A, B). Overall, the number of transcripts related to gibberellin, salicylic acid, cytokinin, and strigolactone were less abundant (Fig. 6A, B ).

The fruit ripening related TFs were similarly distributed in both cultivars (Fig. 6C, D). Transcription factors belonging to the APETALA2/ethylene-responsive element-binding factor (AP2/ERF) and NAC (NAM, ATAF1/2 and CUC2) family were the most abundant followed by homeodomain leucine zipper (HB-zip), basic helix-loop-helix (bHLH), MYB (v-MYB, discovered in avian myeloblastosis virus genome) and MADS-Box family (Fig. 6C, D). Transcription factors encoding the SQUAMOSA promoter binding protein (SBP)-Box and WD40 proteins were the least abundant (Fig. 6C, D).

### ABA and anthocyanin content, and biosynthesis-related gene expression analyses

**Fig. 7:**
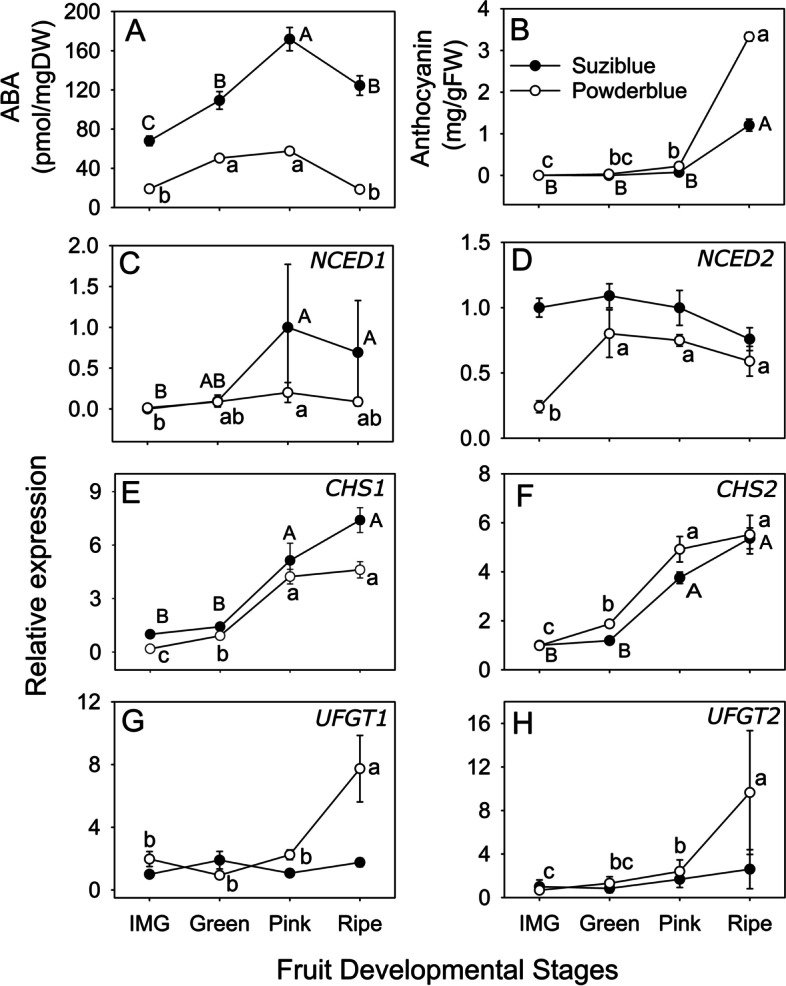
Concentration of ABA (**A**) and anthocyanin (**B**) during fruit ripening in ‘Suziblue’ and ‘Powderblue’. Relative abundance of transcripts involved in ABA biosynthesis (**C, D**) and anthocyanin biosynthesis (**E–H**) during fruit ripening in the two cultivars. Values represent means and standard errors of at least three replicates. ANOVA was used to test for significance (α = 0.05) among stages within a cultivar and means separation was performed using Tukey’s HSD. Means followed by different letter are significantly different with upper and lower case for ‘Suziblue’ and ‘Powderblue’ respectively. *NCED*: *9-CIS-EPOXYCAROTENOID DIOXYGENASE*, *CHS*: *CHALCONE SYNTHASE*, *UFGT*: *ANTHOCYANIDIN 3-O-GLUCOSYLTRANSFERASE*

‘Suziblue’ and ‘Powderblue’ displayed an increase in ABA concentration from Immature Green (IMG) to Pink stage and then decreased during the Ripe stage (Fig. 7A). In ‘Suziblue’, ABA concentrations increased by 1.6-fold between IMG to Green, and Green to Pink stage and then decreased by 1.4-fold between Pink and the Ripe stage (Fig. 7A). In ‘Powderblue’, ABA concentrations increased 2.6-fold between IMG and Green stage, remained similar between Green and Pink stage and then declined 3.1-fold between Pink and Ripe stage (Fig. 7A). The transcriptome database generated in this study was used to identify candidates associated with ABA metabolism and their transcript abundance was measured. Among transcripts associated with ABA biosynthesis, abundance of *9-CIS-EPOXYCAROTENOID DIOXYGENASE1* (*NCED1*) was substantially lower than that of *NCED2* (based on the Ct value; data not shown), suggesting that *NCED2* was the predominantly expressed gene. *NCED1* expression was higher between IMG and Pink stage in both cultivars (Fig. 7C). The pattern of *NCED2* abundance in ‘Suziblue’ was similar across the fruit developmental stages; whereas in ‘Powderblue’ the transcript abundance increased by 3.3-fold between IMG and Green and then remained similar through advanced stages of ripening (Fig. 7D).

ABA application in blueberry promotes anthocyanin biosynthesis and related gene expression [18, 19]. In this study, anthocyanins, accumulated during fruit ripening, especially from Pink to Ripe stage by 16.1-fold and 2.4-fold in ‘Suziblue’ and ‘Powderblue’, respectively (Fig. 7B). Further, multiple anthocyanin biosynthesis genes were identified using the transcriptome and these were further evaluated. *CHALCONE SYNTHASE1* (*CHS1*) and *CHS2* were 4.6- and 2.6-fold higher between Green and Pink stage in ‘Powderblue’ and remained similar between Pink and Ripe stage (Fig. 7E, F). In ‘Suziblue’, *CHS1* and *CHS2* exhibited a similar pattern as mentioned above with a 3.6- and 2.3-fold increase between Green and Pink stage (Fig. 7E, F). Compared to *CHS*, the expression of *ANTHOCYANIDIN 3-O-GLUCOSYLTRANSFERASE* (*UFGT*) was substantially lower (based on Ct value, data not shown). The expression of *UFGT1* and *UFGT2*, increased between Pink and Ripe stage by 3.5- and 4-fold respectively, in ‘Powderblue’; such an increase was not observed in ‘Suziblue’ (Fig. 7G, H).

### Fruit texture and transcript abundance of cell wall modification-related genes during fruit ripening

**Fig. 8:**
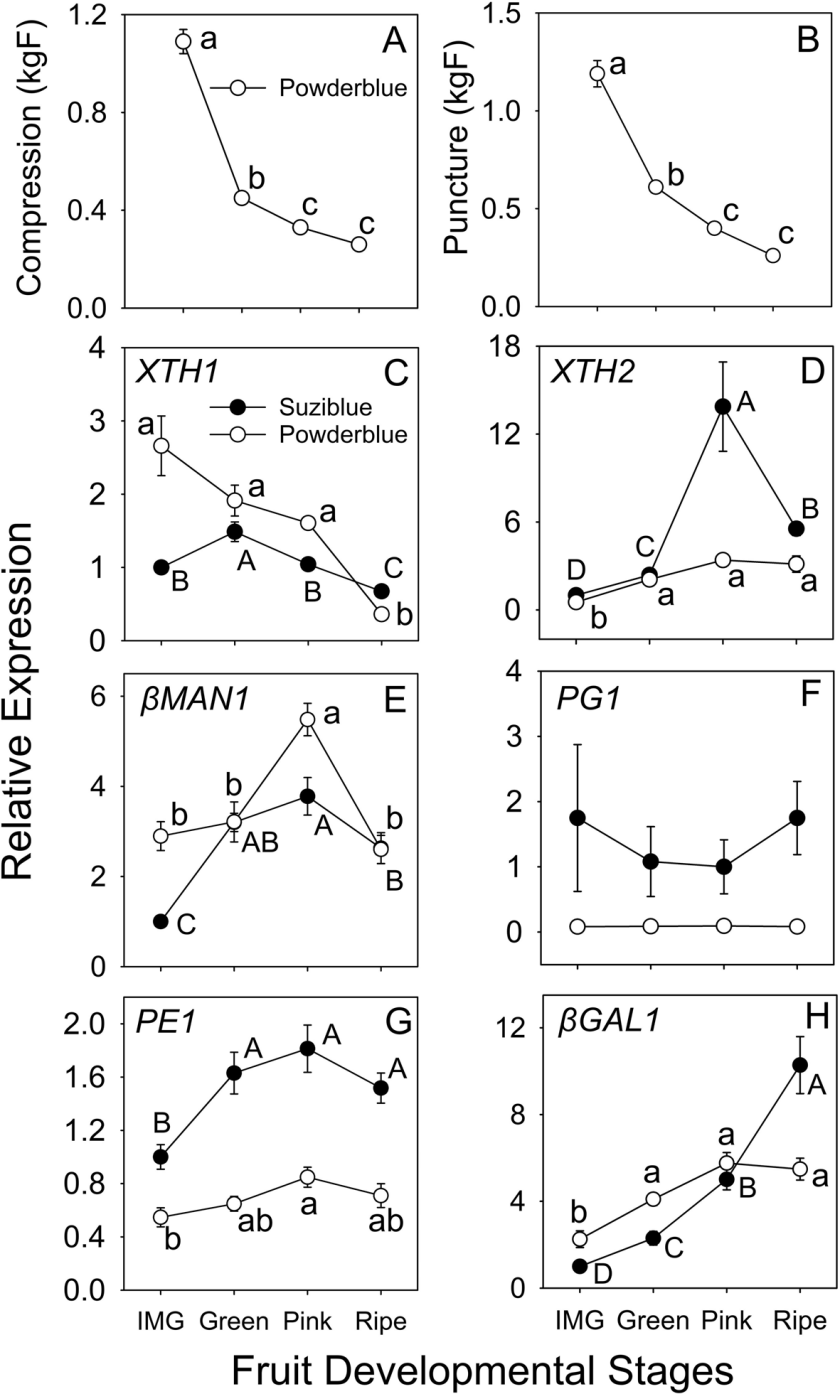
Fruit compression (**A**), puncture (**B**), and relative abundance of transcripts involved in cell wall modification (**C-H**) during fruit ripening in ‘Suziblue’ and ‘Powderblue’. Values represent means and standard errors of at least three replicates. ANOVA was used to test for significance (α = 0.05) among stages within a cultivar and mean separation was performed using Tukey’s HSD. Means followed by different letter are significantly different with upper and lower case for ‘Suziblue’ and ‘Powderblue’ respectively. *XTHI: XYLOGLUCAN ENDOTRANSGLUCOSYLASE/HYDROLASE*, *βMAN*, *1,4-β-MANNOSIDASE, PG*: *POLYGALACTURONASE*, *PE*, *PECTINESTERASE*, *βGAL*: *β-GALACTOSIDASE*

Fruit compression decreased by 2.4- and 1.4-fold, and puncture by 2- and 1.5-fold between IMG and Green, and Green and Pink stages, respectively (Fig. 8A, B). Multiple cell wall remodeling-related transcripts were identified and evaluated. *XYLOGLUCAN ENDOTRANSGLUCOSYLASE/HYDROLASE1* (*XTH1*) abundance decreased during ripening whereas that of *XTH2* increased in both cultivars (Fig. 8C, D). Transcript abundance of *XTH*2 was highest between Green and Pink stage increasing by 5.8-fold and then declining at the Ripe stage in ‘Suziblue’, whereas in ‘Powderblue’, it increased by 6.4-fold between IMG and Pink stage and continued to remain high throughout ripening (Fig. 8D). The expression of *1,4-β-MANNOSIDASE1* (*βMAN1*) increased between the IMG and Pink stages by 3.8-fold and 1.9-fold in ‘Suziblue’ and ‘Powderblue’ respectively, and then declined at the Ripe stage (Fig. 8E). Overall, the transcript abundance of pectin-modifying *POLYGALACTURONASE* (*PG*) was low (data not shown) and its pattern of gene expression did not significantly change during fruit ripening (Fig. 8F). The transcript abundance of *PECTINESTERASE1* (*PE1*) increased by 1.6-fold between IMG and Green in ‘Suziblue’ and then remained constant until the Ripe stage (Fig. 8G). In ‘Powderblue’, *PE1* transcript abundance increased by 1.6-fold only between IMG and Pink stage (Fig. 8G). The expression of *β-GALACTOSIDASE1* (*βGAL1*) increased steadily during ripening in ‘Suziblue’ with a 10.2-fold increase between IMG and the ripe stage, whereas in ‘Powderblue’ it increased by 1.8-fold between IMG and Green and then remained constant until the Ripe stage (Fig. 8H).

## Discussion

The goal of this study was to generate a full-length blueberry fruit transcriptome that can serve as a resource for identifying important ripening, and postharvest-related genes. Further this transcriptome should provide a standalone reference to map short RNA seq-reads, specific to southern highbush and rabbiteye cultivars. The blueberry fruit transcriptome generated a total of 32,119 and 31,323 full-length transcripts in ‘Suziblue’ and ‘Powderblue’ respectively. The number of transcripts were similar to that observed in strawberry PacBio sequencing that generated 33,236 transcripts during fruit development [20]. Of the total unique transcripts, 16,299 and 15,882 non-redundant transcripts were generated in ‘Suziblue’ and ‘Powderblue’, respectively. The haplotype genome suggests 32,140 genes that encode proteins for a given haplotype with a projection of 128,559 total genes for a tetraploid blueberry [21]. ‘Suziblue’, a southern highbush blueberry is tetraploid, while ‘Powderblue’, a rabbiteye blueberry is hexaploid. Blueberry has been suggested to be an allopolyploid and dominance of one of the sub-genomes during various stages of development has been proposed, which has been the case for several allopolyploid species [21]. This may explain the similarity in the number of unique transcripts generated in the tetraploid and hexaploid cultivars used in this study.

The transcriptome assembled by IsoSeq is expected to be full-length and our analyses indicated greater than 80% of transcripts were more than 1,000 nt long. The length on an average ranged between 68 to 7,798 in both cultivars with median transcript length about 1,700 nt. In cotton, transcript length from the full-length transcriptome ranged from 200–10,000 nt [22]. Other studies reported an average length of 2,047 bp [23] and 2,177 bp [24] after full length sequencing. Thus, the results obtained in this study were well within the range obtained from other studies.

The blueberry draft genome generated using 454 and Illumina sequencing predicted about 24% of all the multi-exon genes to display transcript variants due to alternative splicing or promoter use [7]. In this study, 35.96% and 37.12% of non-redundant transcripts in the rabbiteye and the southern highbush cultivars, respectively, had at least one alternative splicing event. The detection of alternative splice events in multi-exon genes has been suggested to be more robust using SMRT sequencing (around 58%) compared with Illumina (around 35%) [20]. Thus, further exploration of alternative splicing events at a large-scale or with certain genes of interest is possible with the current dataset. In the current study, intron-retention was the most common alternative splicing event which was also the case in previous studies [22, 25–29]. However, the frequency of a certain splice event can change depending on the developmental stage. In strawberries intron retention was the most abundant alternative splicing event, however the proportion changed during fruit development with alternative acceptor sites being the major event after fertilization [20]. Splicing patterns have also been suggested to vary according to fruit developmental stages in blueberry [7].

Fruit ripening initiation and progression is coordinated by several hormones. Mainly levels of ABA and ethylene increase, and auxin declines during fruit ripening [30]. Therefore, it was not surprising that in the blueberry fruit transcriptome, among hormone-related categories, the highest number of transcripts were associated with ABA metabolism. A role for ABA in ripening initiation has been proposed in both climacteric fruits such as tomato and non-climacteric fruits such as strawberries and grapes [31–33]. In highbush blueberry, ABA application can promote anthocyanin biosynthesis and transcript abundance of anthocyanin biosynthesis related genes [18, 19]. In blueberries, ABA concentration increases during the onset of ripening after which the levels decline at the Ripe stage [34]. In the current study, ABA concentration was found to increase during ripening until the Pink stage and to decrease between the Pink and Ripe stages, in both cultivars. Overall, the expression patterns of the two transcripts that encode for ABA-biosynthesis genes, *NCED1* and *NCED2* was consistent with ABA production, similar to the results observed in other blueberry studies [18, 34]. Similarly, the expression patterns of two *CHS* and two *UFGT* transcripts coding for enzymes in the anthocyanin biosynthesis pathway reflected changes in anthocyanin concentration. Blueberries have been classified as exhibiting atypical climacteric physiology with a functional ethylene metabolism and signaling during ripening [35]. Further application of ethephon, an ethylene releasing compound can accelerate blueberry ripening [36]. Possibly like in other fruits there may be an interaction among various hormones during ripening in blueberries as well.

During fruit ripening, the coordinated action of many cell wall modification enzymes facilitates the softening process. These enzymes are coded by large gene families, with some members displaying temporal regulation during fruit ripening [22, 37–39]. In this study we selected a subset of cell wall modification enzymes that have important roles during fruit development, including ripening. Consistent with progression of ripening and fruit softening, fruit firmness declined at the initiation of ripening and continued to decrease during its progression. Four transcripts associated with cell-wall modification, *XTH2*, *βMAN1*, *PE1*, and *βGAL1,* displayed an increase in abundance during various periods during ripening, suggesting their importance in facilitating cell wall remodeling during ripening. XTH enzymes display xylogucan transglucosylase and/or hydrolase activity, and are important for fruit softening, for example in persimmon [40, 41]. Of the two *XTH* transcripts characterized in this study only *XTH2* expression increased during fruit ripening. The transcript abundance of *XTH1* was higher in the IMG fruit suggesting its role in cell wall modification during early fruit development. Such a temporal regulation for *XTH* gene family members have been previously reported in tomato [42]. Another enzyme similar to XTH in function, βMAN, displayed high activity during fruit ripening in tomato [43, 44]. Similarly, the *βMAN1* transcript selected in this study exhibited higher transcript abundance at the Pink stage*.* In addition, pectin modifying enzymes such as PG, PME and βGal play a positive role in fruit softening during ripening and postharvest storage [45–48]. In this study *PME1* and *βGal1* were induced during blueberry ripening, however the transcript abundance of *PG1* was low in the southern highbush cultivar and almost undetectable in the rabbiteye cultivar. PG activity in highbush blueberry is high during initial ripening stages and then declines as ripening progresses and is correlated with pectin depolymerization [49]. While the current study does not support such a role in southern highbush and rabbiteye blueberry, it is possible that ripening specific *PGs* were not targeted for gene expression analysis here.

Upstream of ripening-related genes, transcription factors such as NAC, MADS-box, and CNR play a key role in ripening in tomato [50]. Multiple potential ripening-related transcription factors were identified from the full-length transcriptome. NAC transcription factors play a role in ripening and in leaf and fruit senescence [51–54]. One of the well-studied members of the NAC transcription factor family, NON-RIPENING (NOR), and another member NOR-like 1 positively regulates ripening upstream of ethylene [50–52, 55]. More recently, NAC transcription factors were shown to downregulate the transcript abundance of the abscisic acid biosynthesis gene, *NCED* in citrus, suggesting a potential negative role for some members within this family [56]. In tomato, RIN, a MADS-box transcription factor, and CNR, a member of the SBP-box gene family are important in ripening initiation [57–60]. In addition, the transcriptome also contained anthocyanin-related transcription factors. Transcriptional regulation of anthocyanin biosynthesis is coordinated via a protein complex consisting of MYB, bHLH, and WD40 [61]. This complex was also shown to regulate anthocyanin biosynthesis in blueberry fruit [62].

Collectively, the expression of ABA and anthocyanin biosynthesis related genes, and cell wall modification genes indicate that the current transcriptome is robust and offers full length gene sequences for investigating molecular aspects of ripening and postharvest processes.

## Conclusions

This study describes the first full-length fruit transcriptome for southern highbush and rabbiteye type of blueberry. This transcriptome is a useful resource and can be used as a standalone reference for mapping short RNA seq-reads specific to southern highbush and rabbiteye blueberry cultivars. Further, this study demonstrates the robustness of the transcriptome with respect to identification of genes associated with fruit ripening and quality traits. Together, the full-length transcriptome developed here offers a valuable genomic resource that can help to facilitate breeding of fruit quality and other related traits in blueberry.

## Methods

### Sample collection

Fruit from southern highbush ‘Suziblue’ (*Vaccinium sp.*) and rabbiteye blueberry ‘Powderblue’ (*V. virgatum*) were collected at various stages of ripening and postharvest storage. Fruit used in this study were from cultivated species and appropriate permissions were obtained for fruit collection. All experimental procedures including use and collection of the fruits comply with the ethical standards and legislations. Ripening stages included Immature Green (IMG), Green, Pink, and Ripe. IMG stage was defined as a combination of S4 (7–9 mm) and S5 (9–13 mm) based on size [34]. Green stage was based on size and color (< 13 mm or < 30% pink skin). The remaining two stages were harvested based on surface coloration with Pink stage being predominantly pink (> 50%) and Ripe fruit being fully blue. Fruit from these stages were frozen in liquid N_2_ immediately after harvest in the field and stored at -80 °C. To collect fruit at various postharvest stages, ripe fruit were hand harvested and stored in 1-pint clamshells in a walk-in cooler at 4 °C and approximately 90% relative humidity. Fruit were taken at three stages during storage, left at room temperature (~ 25 °C) for 2 h, subsequently frozen in liquid N_2_ and stored at -80 °C until further use. The three postharvest (PH) stages of ‘Suziblue’ were 3 d after storage (PH3), 8 d after storage (PH8) and 13 d after storage (PH13), and for ‘Powderblue’ PH4, PH8, and PH14 (4, 8 and 14 d after storage). For ‘Suziblue’ all fruit were collected from the UGA Blueberry Research farm in Alapaha, GA (31.345288, -83.240317) in 2015, except for the IMG stage collected from the same farm in 2018. All ‘Powderblue’ fruit were collected from the Durham Horticulture Research farm in Watkinsville, GA (33.8872879, -83.4206120) in 2018. In each cultivar, for every stage multiple fruit (~ six) were pooled for RNA extraction.

### RNA extraction and PacBio iso-seq

All samples were ground into a fine powder using a mortar and pestle with liquid N_2_ for RNA extraction. RNA extraction was performed by using a modified CTAB-based method [63]. RNA was analyzed on a Bioanalyzer (Agilent Technologies, 2100, CA) to ensure high quality of RNA (RIN > 8). For each cultivar, 2 µg of RNA from all developmental and postharvest stages described above were pooled. Libraries of the two samples were constructed following Pacific Biosciences’s standard protocol for Iso-Seq (Iso-Seq Template Preparation for Sequel Systems). Each library was sequenced on 1 SMRT cell on a PacBio Sequel Systems (Pacific Bioscience, CA).

### Transcriptome reconstruction and annotation

The full-length transcripts were characterized by the isoseq3 tool in SMRT Link v8.0, a software designed by PacBio to analyze Single Molecule, Real-Time (SMRT) sequencing data, with the following steps suggested by the SMRT Tools Reference Guide: 1) circular consensus sequences (CCS) were generated from the raw data sub-reads. 2) the IsoSeq v2 primer sets at both ends of the sequences were removed. 3) noises including poly A tails and concatemers were removed generating full-length, non-concatemer (FLNC) reads. 4) full-length unpolished transcripts were generated by clustered consensus sequences. 5) full-length unpolished transcripts were polished by using raw subreads, including non-full-length transcripts, to generate full-length transcripts (Fig. 1). Next to remove the redundancy, the polished full-length transcripts were mapped to the northern highbush blueberry ‘Draper’ (*V. corymbosum*) genome [21] and then collapsed transcripts by genomic mapping. The transcripts unmapped to the genome were collapsed by Cogent (https://github.com/Magdoll/Cogent/). Finally, non-redundant transcripts were generated after combining the transcripts collapsed after mapping to the genome and those from Cogent analyses (Fig. 1).

The non-redundant transcripts were further annotated by Blast2GO [64]. The annotation process included 1) performing Blastx against NCBI non-redundant protein database with flowering plants as taxonomy filter, 2) retrieving domain/motif information by InterProScan, and 3) assigning gene ontology (GO) terms and enzyme codes. Except for the parameters mentioned above, all steps were performed with the default settings.

### Alternative splicing identification and long non-coding RNA analysis

The generic feature format output generated by Isoseq3 and Cogent were used to classify alternative splicing events by alternative splicing transcriptional landscape visualization tool (AStalavista) [65]. To identify long non-coding RNA, the non-redundant transcriptomes for each cultivar were analyzed by Coding-Non-Coding Identifying tool (CNIT) [66] with the setting of the plant mode (Fig. 1).

### Identification of phytohormone-related genes and ripening-related transcription factors

For phytohormone-related genes, the GO category for biological processes was searched using key words for hormones which included, ABA, auxin, brassinosteroid, jasmonic acid, ethylene, gibberellin, salicyclic acid, cytokinin, and strigolactone (Additional file 1: Table S4, S5). For mining of transcription factors, sequences were retrieved from the Sol Genomics Network (https://solgenomics.net/) for tomato and from NCBI (https://www.ncbi.nlm.nih.gov/) for strawberries. The transcription factors belonged to various gene families with known roles in ripening. The tomato MADS-Box genes included, *RIPENING INHIBITOR* (*RIN*: Solyc05g012020), *TOMATO* *AGAMOUS-LIKE 1* (*TAGL1*: Solyc07g055920), *MADS1* (Solyc03g114840), *FRUITFUL1* (*FUL1*: Solyc06g069430), and *FRUITFUL2* (*FUL2*: Solyc03g114830), and from strawberry, MADS-RIN (AF484683.1). Also, transcription factors belonging to other families such as, NAC (*NAC4*: Solyc11g017470, *NOR*: Solyc10g006880), *AP2/ERF* (*AP2*: Solyc03g044300; *ERF*: Solyc01g065980), SBP-Box (*SPL-CNR*: Solyc02g077920), and *HB-zip* (*HB1*: Solyc02g086930) were identified from tomato. Transcription factors related to anthocyanin biosynthesis included the MYB (JQ989281), bHLH domain protein (JQ989284), and WD40 protein (JQ989287) from the strawberry. These transcription factors were used to identify blueberry homologs in the ‘Powderblue’ and ‘Suziblue’ transcriptomes using BLAST analysis (tblastx function). For all the blueberry homologs identified, reciprocal blast analysis was performed to confirm their gene identity.

### ABA quantification

For ABA quantification, 0.6 g of fine powdered frozen fruit sample was shipped in dry ice to the University of North Texas (UNT) BioAnalytical Facility. The protocol used by UNT was based on [67]. Briefly, samples were lyophilized, and 10 mg of sample extracted with 400 µL of 10% methanol containing 1% acetic acid with an internal standard, ^13^C-benzoic acid. Subsequently samples were disrupted using a Tissuelyzer bead mill, and then incubated in ice for 30 min. Samples were then centrifuged at 17,000 × *g* for 10 min at 4 °C and the supernatant collected. The pellet was re-extracted in methanol and acetic acid and the two supernatants pooled. The sample was filtered using ultra-centrifugal filter units (Amicon, Florida, USA). Finally, ABA was quantified by liquid chromatography with tandem mass spectrometry (LC–MS/MS).

### Anthocyanin measurements

Fruit tissue was finely ground in liquid N_2_ using a mortar and pestle. 100 mg of ground sample was extracted using 2 mL of 100% methanol acidified with 0.1 mL HCl [68]. Samples were mixed briefly using a vortex and sonicated in dark for 10 min using a Bransonic 220 sonicator (Branson Ultrasonics Corp., Danbury, CT). Next, tubes were mixed in the dark for 1 h at 300 ppm using a Environ-shaker (Lab-Line Instruments Inc., Melrose Park, IL) and centrifuged at 2000 × *g* for 25 min at 20 °C. The supernatant was transferred into a new tube. Total anthocyanins were measured using the differential pH method. 40 µL of the supernatant along with 160 µL of 0.025 M potassium chloride (pH 1.0) and 160 µL of 0.4 M sodium acetate (pH 4.5) was transferred into a 96 well plate (Becton Diskinson, Franklin Lakes, NJ). A blank was prepared using 40 µL of extraction buffer (methanol acidified with HCl) along with the two buffers described above. The samples were mixed with the buffers using a multichannel pipette and the absorbance measured at 520 and 700 nm using a Biotek microplate reader (BioTek, Winooski, VT). Anthocyanin concentration was calculated as cyanidin-3-glucosides equivalents using the equation in [69, 70].

### Fruit firmness measurements

Fruit compression and puncture in ‘Powderblue’ were measured as described in [36]. Briefly, 12 fruit per replicate (total four replicates) were used for compression and puncture analyses using a fruit texture analyzer (GS-15, Güss Manufacturing, Strand, South Africa). For compression analyses, a probe with a 15-mm diameter end plate was used to calculate the force required to compress the fruit by 1 mm. For puncture analyses, a 1.5-mm probe was used to measure the force required to puncture the fruit skin while traveling a distance of 3 mm.

### Gene expression analysis

Four fruit developmental stages, IMG, Green, Pink, and Ripe stages were used for gene expression analyses. Fruit samples from the cultivar, Suziblue, were the same as that used for IsoSeq. For the cultivar, Powderblue, samples harvested in 2020 as previously described from the Durham Horticulture Research farm in Watkinsville, GA. The RNA extraction method was the same as described above. The synthesis of cDNA was conducted using 1 µg of total RNA. After removing any potential DNA from the RNA sample by DNase treatment, cDNA was synthesized by reverse transcription following the manufactures’ protocol (Promega, Madison, WI, USA) and the final volume was diluted to 100 µl. After that, real-time quantitative reverse transcription PCR (qRT-PCR) analysis was performed by using 1 μL of cDNA with PowerUp SYBR Green Master Mix reagent (Applied Biosystems, Foster City, CA, USA) and with Stratagene Mx3005P qRT-PCR instrument (Agilent Technologies, USA) to measure the expression of the following genes: *NCED*, *CHS*, *UFGT*, *XTH*, *PG*, *PE*, and *βGAL* (Additional file 2). Three reference genes, *UBIQUITIN-CONJUGATING ENZYME* (*UBC28*), *RNA HELICASE-LIKE* (*RH8*), and *CLATHRIN ADAPTER COMPLEXES MEDIUM SUBUNIT FAMILY PROTEIN* (*CACSa) *[63], were used to normalize the expression of the target gene. The concentration of forward and reverse primers was 0.2 μM for all genes except *PG* (0.15 μM). The reaction conditions were set at: 50 °C for 2 min, 95 °C for 5 min, followed by 95 °C for 15 s, and 60 °C for 1 min, repeated for 40 cycles for all genes, except *NCED*. For *NCED*, the annealing and extension temperatures were set at 64 °C. Melt curve analysis was performed at 95 °C for 1 min, 55 °C for 30 s, 95 °C for 30 s to check for primer specificity. Efficiency of the qRT-PCR reactions were determined using the LinRegPCR software [71]. The relative quantity (RQ) values were determined using the Ct (threshold cycle) value and were corrected for primer efficiency [72]. RQ values of a given sample were normalized using the geometric mean of the RQ values of reference genes for that sample to determine normalized RQ values (NRQ) [72]. The Pink Stage was used as the reference stage for *NCED1* and *PG* and all data are expressed as fold-change in relation to gene expression at this stage. For all other genes, the IMG stage of ‘Suziblue’ was set as the reference stage, with fold-change for gene expression calculated using this stage. Standard error was calculated as described in [72]. Statistical analyses were performed on log_2_ transformed NRQ values. Statistical analysis was performed using one-way analysis of variance (ANOVA) to compare across stages within a given genotype. If significant (*P* ≤ 0.05), mean separation was performed using Tukey’s honestly significant difference (HSD) test in JMP Pro 15 (SAS Institute, Cary, NC, USA). All figures were made using SigmaPlot (ver. 14.0; SYSTAT, Palo Alto, CA, USA) and figures were compiled using Inkscape (ver. 1.0; Boston, MA, USA).

## Supplementary Information

**Additional file 1:** **Table S1.** The annotation of fruit-specific transcriptome in Suziblue. **Table S2.** The annotation of fruit-specific transcriptome in Powderblue. **Table S3.** List of long non-coding RNA in fruit-specific transcriptomes of Suziblue and Powderblue. **Table S4.** Summary of hormone-related transcripts in Suziblue fruit-specific transcriptome. **Table S5.** Summary of hormone-related transcripts in Powderblue fruit-specific transcriptome.

**Additional file 2.** List of primers used for QRT-PCR analysis.

## Data Availability

Datasets used in the current study that support the conclusion are included within the article and as additional files. The raw reads are available in the NCBI Sequence Read Archive (SRA) BioProject database (BioProject ID PRJNA814709; http://www.ncbi.nlm.nih.gov/bioproject/814709). The link provided will be active upon acceptance of this manuscript. Other data sets generated in this study will be available from the corresponding author upon request.

## Abbreviations

ABA: Abscisic acid
AP2/ERF: APETALA2/ethylene-responsive element-binding factor
bHLH: Basic helix-loop-helix
*CACSa*: *CLATHRIN ADAPTER COMPLEXES MEDIUM SUBUNIT FAMILY PROTEIN*
*CHS*: *CHALCONE SYNTHASE*
CCS: Circular consensus sequences
CNIT: Coding-non-coding identifying tool
*FUL1*: *FRUITFUL1*
*FUL2*: *FRUITFUL2*
FLNC: Full-length, non-concatemer
GO: Gene ontology
HB-zip: Homeodomain leucine zipper
IMG: Immature green
Iso-Seq: Isoform sequencing
LC–MS/MS: Liquid chromatography with tandem mass spectrometry
*NAC*: *NAM*, *ATAF1/2* And *CUC2*
*NCED*: *9-CIS-EPOXYCAROTENOID DIOXYGENASE*
*NOR*: *NON-RIPENING*
nt: Nucleotides
PacBio: Pacific biosciences
*PE*: *PECTINESTERASE*
*PG*: *POLYGALACTURONASE*
PH: Postharvest
qRT-PCR: Real-time quantitative reverse transcription PCR
*RH8*: *RNA HELICASE-LIKE*
*RIN*: *RIPENING INHIBITOR*
SMRT: Single molecule, real-time
SBP: SQUAMOSA promoter binding protein
*TAGL1*: *TOMATO* *AGAMOUS-LIKE 1*
*UBC28*: *UBIQUITIN-CONJUGATING ENZYME*
*UFGT*: *ANTHOCYANIDIN 3-O-GLUCOSYLTRANSFERASE*
*XTH*: *XYLOGLUCAN ENDOTRANSGLUCOSYLASE/HYDROLASE*
*βGAL*: *β-GALACTOSIDASE*
*βMAN*: *1,4-β-MANNOSIDASE*
