# Supplementary figures and images for: Full-length fruit transcriptomes of southern highbush (Vaccinium sp.) and rabbiteye (V. virgatum Ait.) blueberry
Source: BMC Genomics. 2022 Oct 29;23:733. doi: 10.1186/s12864-022-08935-5 (PMC9618223; doi:10.1186/s12864-022-08935-5)

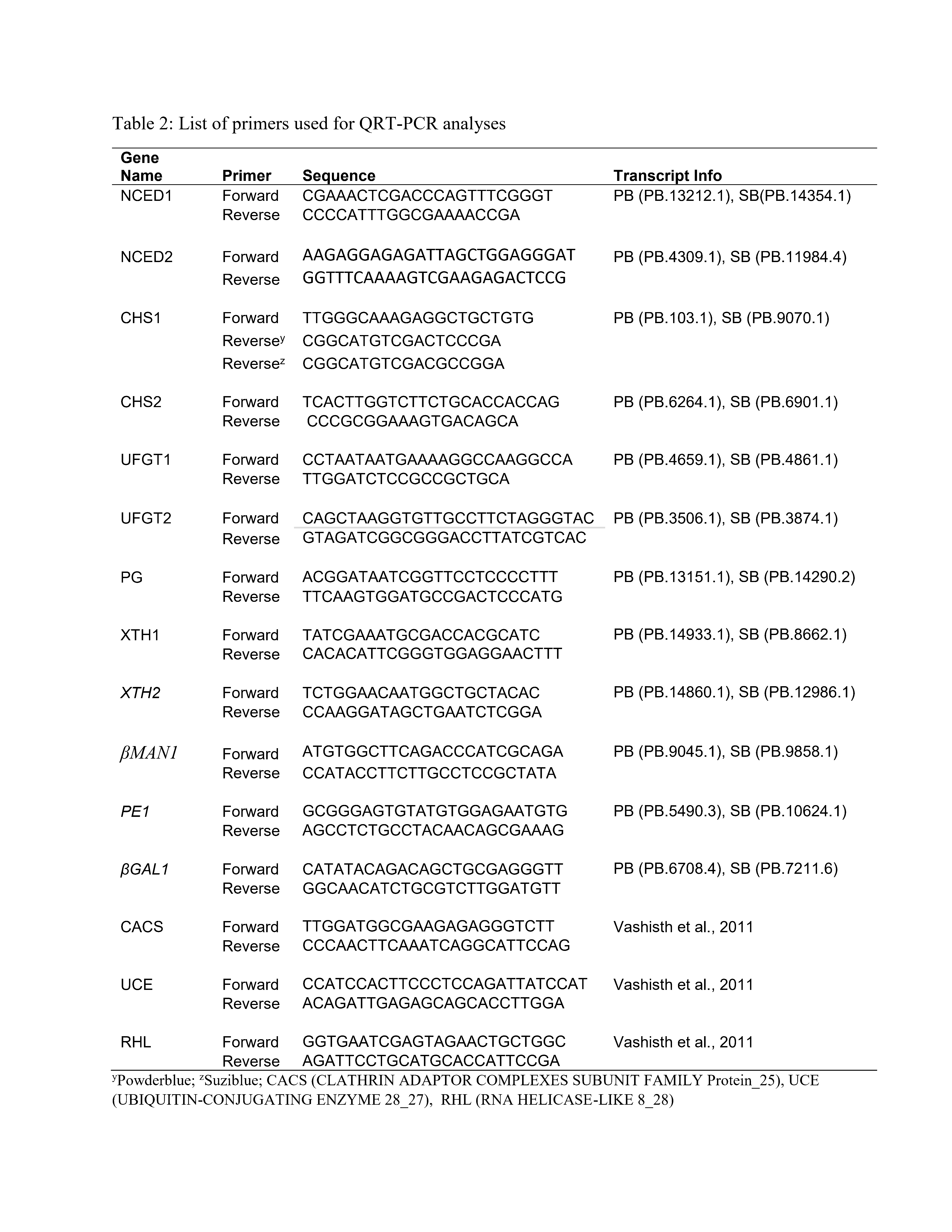

Supplement: Supplementary file 2 — Additional file 2. List of primers used for QRT-PCR analysis. [file 12864_2022_8935_MOESM2_ESM.tiff]
